# Pre-induction translabial ultrasound measurements in predicting mode of delivery compared to bishop score: a cross-sectional study

**DOI:** 10.1186/s12884-016-1090-x

**Published:** 2016-10-28

**Authors:** Soghra Khazardoost, Fahimeh Ghotbizadeh Vahdani, Sahar Latifi, Sedighe Borna, Maryam Tahani, Mohammad Ali Rezaei, Masoomeh Shafaat

**Affiliations:** 1 Department of obstetrics and gynecology, Vali-Asr hospital, Tehran University of Medical Sciences, Vali-Asr hospital, Imam Khomeini Hospital Complex, Keshavarz Boulevard, Tehran, 1419733141 Iran; 2 Tehran University of Medical Sciences, Tehran, Iran; 3 Maternal, Fetal & Neonatal Research Center, Vali-Asr hospital, Tehran University of Medical Sciences, Tehran, Iran

**Keywords:** Ultrasound, Cesarean section, Bishop

## Abstract

**Background:**

By increased concerns about the accuracy of the traditional methods to predict outcomes after induction of labor, developing new standards has a great clinical importance. Here, we compared the predictive value of translabial ultrasound measurements with Bishop Score to determine the suitability of induction of labor.

**Methods:**

A homogenous population of primigravid women was recruited. Induction of labor was performed with low-dose infusion of oxytocin. Translabial ultrasound and assessment of Bishop Score were performed by two different obstetricians. Receiver–operating characteristics curves were obtained to measure area under curve and subsequently, test sensitivity of each method.

**Results:**

One hundred women entered the investigation. Maternal body mass index was significantly higher among candidates of Cesarean section (P: 0.02). Maternal age and fetus weight, gender and occiput position were not determinants of outcomes of induction of labor. Cervical length and fetal head-pubis symphysis distance measured by translabial ultrasound had a test sensitivity of 90 and 88 %, respectively which were slightly higher than sensitivity of Bishop score (84 %).

**Conclusion:**

This study demonstrates that translabial measurements can be a suitable alternative method to monitor labor progress with an admissible predictive value compared with Bishop Score. It is a non-invasive method which provides valuable objective measurements and can be better accepted by women when considering the painful process which is required in evaluating Bishop Score.

## Background

Although induction of labor (IOL) frequently occurs in term pregnancies, it has been demonstrated that it can be associated with higher rate of Cesarean section (C/S) [1, 2]. Therefore, many investigations have tried to determine factors which are related to a successful vaginal delivery after IOL [3]. Fetal distress is the most common reason for necessity of an operative delivery when IOL fails [4]. Identifying factors that can predict the success of IOL is clinically essential. Previously, Bishop Score has been used as the ‘gold standard’ predicting the suitability of IOL [5]. Bishop Score is measured by assessment of dilatation, effacement, consistency and position of the cervix and fetal station [5]. Since Bishop Score is a subjective measure, it can be accompanied with high intra- and inter-observer variability [6–11]. Moreover, the procedure of calculating Bishop Score is painful. By considering the limitations of Bishop Score the necessity of identification alternative measures to predict suitability of IOL is clear.

Previously, Newman et al. [12] showed that Bishop Score predictive value is lesser than Cervical score at 26–29 weeks of pregnancy. Furthermore, some studies have shown that transvaginal ultrasound measurements perform better that Bishop Score [13–16]. However, conflicting results exist when addressing the comparison between Bishop Score and Ultrasound measurements [17, 18]. Existence of these conflicting results emphasize on more research in this field especially with adjustment for other confounders to come to a proper comparison. Eggebø et al. [3] demonstrated that the predictive value of fetal head–perineum distance measured by trans-perineal ultrasound is similar to Bishop Score. However, since other factors such as parity and body mass index (BMI) are known to affect prediction of vaginal delivery after IOL [3], investigations on homogenous populations with considering the confounders were required to compare the predictive value of Bishop Score and ultrasound measurements.

In this study, only primigravid women were included and the predictive values of Bishop Score and translabial ultrasound measurements in determining suitability of IOL have been evaluated. Translabial ultrasound has been shown to be a suitable technique to assess labor [19].

## Methods

### Study design and participants

Participants were admitted women with term pregnancies in Tehran University Hospital. Inclusion criteria were: primigravity, term pregnancy with gestational age > 37, healthy fetus with no detected anomalies, singleton fetus with cephalic presentation. Exclusion criteria were pre-term pregnancies, previous Cesarean section or other uterine surgeries, twin fetuses (multiple pregnancy), any suspicious finding of fetal distress at the time of admission. Women with gestational diabetes or suspicious findings indicating fetus macrosomia were excluded and only women with relatively similar range of fetus weight according to previous ultrasound examinations were recruited. Women with cephalopelvic disproportion detected in previously performed examinations were excluded as well. Total of 100 women were recruited in this prospective study. Written consent was obtained from each individual. Participation in the study was voluntary and those women with unwillingness to participate were considered as not eligible. Data was collected between 2012 and 2013.

The gestational age was determined based on the date of the last menstrual period and ultrasound measurements before 16 weeks of gestation. Labor arrest was defined based on Williams’ Obstetrics criteria as arrest in cervical dilatation and fetus descends [20, 21].

### Induction of labor (IOL)

Labor was induced with low-dose oxytocin. The infusion of oxytocin was started at 1 mili unit per minute and was increased by 1–2 mili units per minute every 20 min until adequate uterine contraction was obtained [22]. Amniotomy was performed if the cervix was favorable (Bishop Score ≥6). Successful IOL was defined as vaginal delivery regardless of the required time for its occurrence. Similar dosage and method were applied for all women to induce labor. For those patients with Bishop Score < 5, 25 mg Misoprostol was administered before IOL.

### Translabial ultrasound measurement

Translabial Ultrasound was performed by using a Siemens ultrasound system with a five megahertz curved array transducer. The probe was positioned translabialy along with the following anatomical structures [23]: the pubic symphysis joint and the fetal skull. The transducer was placed in a way so that the symphysis was in horizontal position. All Ultrasound measurements were performed immediately after emptying the bladder and in supine position.

**Fig. 1:**
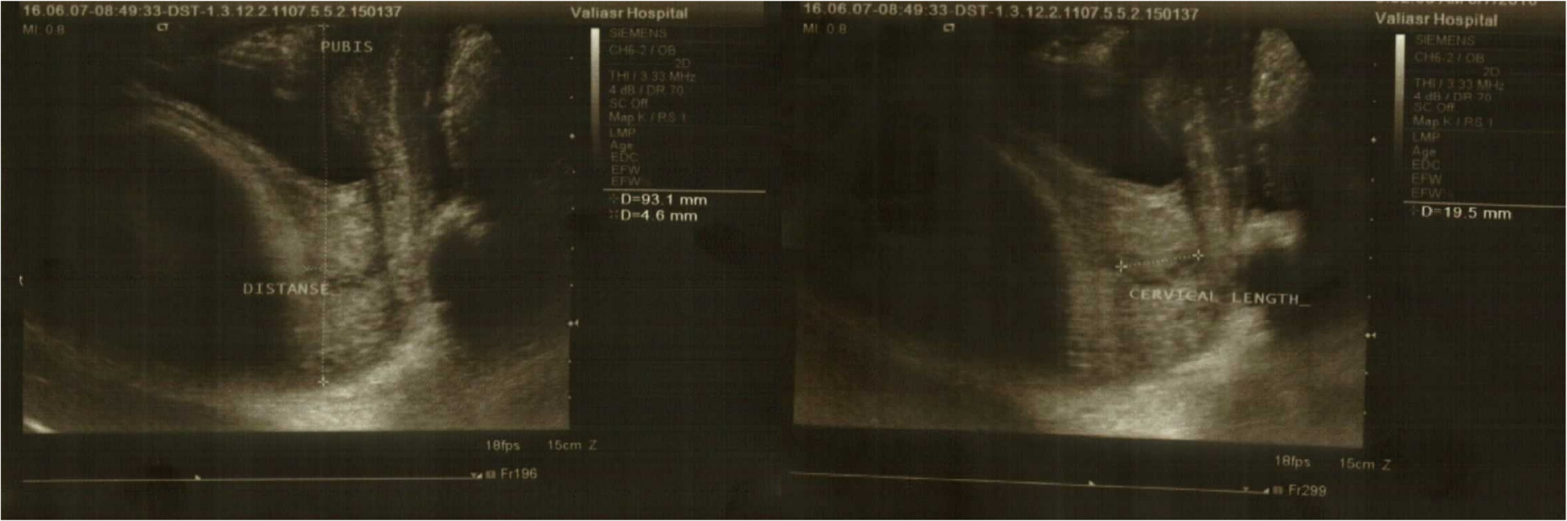
Cervical length and fetal head-symphysis pubis distance as measured in sonographic images

The fetal head–perineum distance was defined as the shortest distance between a line through the inferior posterior symphyseal margin (parallel to the main transducer axis) and the leading edge of the fetal skull. This measure is the distance from the outer bony limit of the fetal skull to the skin surface of the perineum [24]. Negative values were given when the presenting part was found cranial to the line of reference. Positive values imply that the head was seen beyond this line. Figure 1 shows the sonographic images in measuring cervical length and fetal head-symphysis pubic distance. Fetal head-pubis symphysis distance was measured according to the method described by Dietz et al. [23] Cervical length was measured by the same probe and at the same position, without any pressure to soft tissue [25]. Fetus entry angle and occiput position (anterior, transverse and posterior) were determined as well.

### Bishop score

The Bishop score was assessed after performing ultrasound and immediately before IOL by another obstetrician who was blinded to the ultrasound measurements. Scoring was as follows: Position of cervix (posterior: 0, intermediate: 1, anterior: 2), consistency of the cervix (firm: 0, intermediate: 1, soft: 2), effacement (0-30 %:0, 31-50 %: 1, 51-80 %: 2, >80 %: 3), dilation (0 cm: 0, 1-2 cm: 1, 3-4 cm: 2, >5 cm: 3), fetal station (−3: 0, −2: 1, −1 and 0: 2, +1 and +2: 3). A score ≤ 5 suggests that labor is unlikely to start without induction. A score ≥ 9 indicates that labor will most likely commence spontaneously [26].

### Statistical analysis

All the statistical analysis was performed using SPSS version 21 (IBM corporation, USA). Categorical data are presented with percentages and continuous variables are presented with mean ± standard deviation (SD). Comparison of categorical variables between groups (NVD and C/S) was performed using Pearson Chi-square and Fisher’s Exact tests. One-way analysis of variance (ANOVA) was performed to compare means between groups. Partial correlation analysis with controlling for and BMI was used to assess the relationship between continuous variables. Multivariate analysis with adjustment for BMI was performed to assess the differences in continuous variables between groups (NVD vs. C/S). The predictive value of ultrasound measurements and Bishop Scores for a successful vaginal delivery was evaluated using receiver–operating characteristics (ROC) curves in which the area under the curve is used as discriminator to test the diagnostic performance of certain markers [3]. *P* < 0.05 was considered statistically significant.

## Results

One hundred women with mean age of 25.1 ± 4.4 years were enrolled. Table 1 shows the baseline characteristics of participants. Eighty nine women experienced the active phase of labor after IOL but only 57 participants had NVD and 43%of patients needed Cesarean section (C/S). Eleven participants didn’t enter the active phase and no changing was detected in the cervical dilatation progress, despite the adequate contraction of uterus. The indications of C/S were fetal distress, thick meconium and non-favorable labor progression. Arrest in labor progression was identified as arrest in any stages even with a full cervical dilatation.

**Table 1:** Baseline maternal and fetal characteristics among participants with term pregnancy who were candidate for induction of labor

Category		Number (percentage)	Mean (SD)
Age (years)			25.13 (4.4)
Initial weight (kg)			63.69 (10.3)
Initial BMI (kg/m^2^)			24.12 (3.4)
Weight at the time of IOL (kg)			76.04 (1.1)
BMI at the time of IOL (kg/m^2^)			28.80 (3.7)
Cervical length (mm)			20.60 (6.6)
Fetus weight (gr)			3334 (393.6)
Fetus gender	Male	46 (46 %)	
	Female	54 (54 %)	
Bishop score	≤5	77 (77 %)	
	>5	23 (23 %)	
Time between IOL and initiation of contractions (hour)			2.0 (1.1)
*Time between IOL and initiation of cervical dilation (hour)			2.14 (0.8)
*Time between initiation of cervical dilation to 4 cm dilation (hour)			4.12 (1.6)
*Time between cervical dilation of 4 cm to 10 cm (hour)			4.60 (1.5)
*Time between full cervical dilation to labor (hour)			3.12 (2.7)
Delivery	NVD	57 (57 %)	
	C/S	43 (43 %)	

*BMI* Body Mass Index, *C/S* Cesarean Section, *IOL* Induction of labor, *NVD* Normal vaginal delivery, *SD* Standard Deviation. *Time intervals between IOL and cervical dilations (to 4 cm and then to 10 cm) are only measured for patients who had vaginal delivery. In patients who had inadequate progression of cervical dilation, C/S was performed

BMI before pregnancy and BMI at the time of IOL were significantly higher among those women who were candidate for C/S (P: 0.04 and 0.02, respectively). There was no significant difference in the age between women with NVD and those who needed C/S (P: 0.19). Bishop score was ≤ 5 in 77 % of term pregnancies (Table 1). Fetus gender and weight did not differ between NVD and C/S groups (P: 0.24 and 0.19). These outcomes showed that BMI can be considered as a factor which affects the progress of delivery whereas mode of delivery is not influenced by age, fetus gender and weight. Therefore further analyses were performed with adjustment for BMI.

Higher cervical length was detected among women who were candidate for C/S (P: 0.037). As expected, women with successful IOL had higher Bishop Score (P: 0.03). Another factor that determined the suitability of IOL was fetal head-pubis symphysis distance (+3.00 ± 2.5 and −7.2 ± 0.66 in NVD and C/S, respectively; P: 0.019) (Table 2). Apgar scores in all deliveries were 9 or 10. Among women who had successful vaginal delivery, fetus occiput position was anterior in 36.8 %, transverse in 47.4 % and posterior in 15.8 %. Similarly, fetus occiput position was anterior in 28 %, transverse in 46.5 % and posterior in 25.5 % among candidates of C/S (Table 2). The fetus entry angle and the occiput position were not related to the type of delivery (P: 0.05 and 0.41, respectively).

**Table 2:** Comparison of maternal and fetal factors between women with normal vaginal delivery after induction of labor and those who were candidate for Cesarean section. Analysis of the mean difference of cervical length, Bishop Score and fetal head-pubis symphysis distance was performed with multivariate analysis with adjustment for body mass index

Category		NVD (n: 57)		C/S (n: 43)		*P*-value
		Number (Percentage)	Mean (SD)	Number (Percentage)	Mean (SD)	
Age (years)		-	25.66 (4.74)	-	24.47 (4.0)	0.19
Initial BMI (kg/m^2^)		-	23.07 (3.26)	-	24.91 (3.57)	0.04*
BMI at the time of IOL (kg/m^2^)		-	28.07 (3.46)	-	29.78 (3.81)	0.02*
Fetus weight (gr)		-	3378.9 (306.9)	-	3274.53 (483.1)	0.19
Fetus gender	male	24 (42.1 %)	-	22 (51.2 %)	-	0.24
	female	33 (57.9 %)	-	21 (48.8 %)	-	
Cervical length (mm)		-	19.49 (0.12)	-	22.08 (7.11)	0.03*
Bishop Score		-	4.63 (1.55)	-	3.93 (1.60)	0.01*
Fetal head-pubis symphysis distance		-	+3.00 (2.5)	-	−7.2 (0.66)	0.01*
The fetus entry angle		-	90.10 (9.18)	-	85.04 (11.0)	0.05
Fetus occiput position	Anterior	21 (36.8 %)	-	12 (28 %)	-	0.41
	Transverse	27 (47.4 %)	-	20 (46.5 %)	-	
	Posterior	9 (15.8 %)	-	11 (25.5 %)		

*BMI* Body mass index, *C/S* Cesarean section, *IOL* induction of labor, *NVD* Normal vaginal delivery, *SD* Standard deviation

* Significance at the level of *P* < 0.05

The ROC curve showed an Area Under Curve (AUC) of 0.65 (*P* = 0.01) for fetal head-pubis symphysis distance and subsequently the sensitivity and specificity were measured to be around 88 and 70 %, respectively with cut off 12 mm for prediction of NVD.

**Fig. 2:**
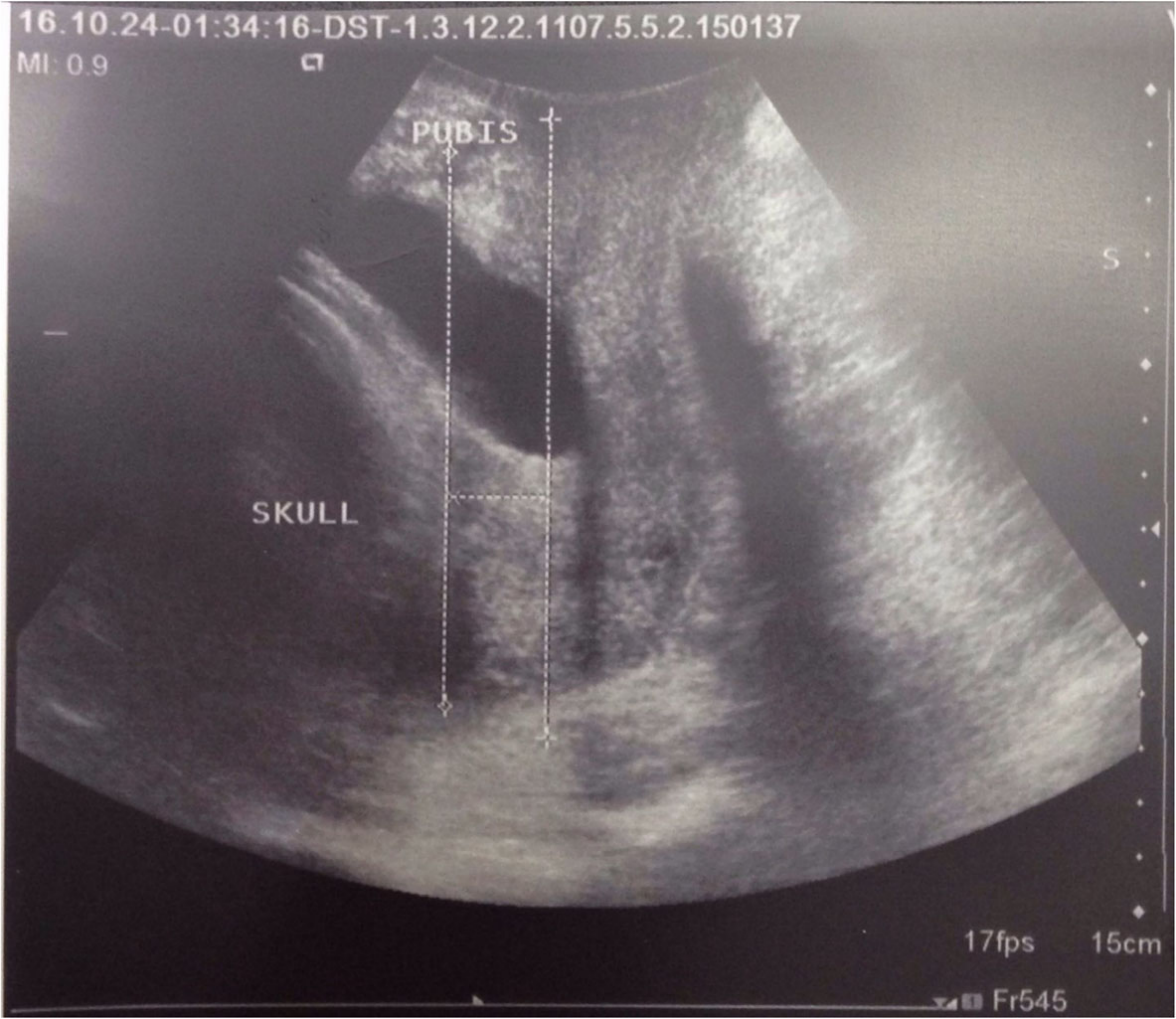
Receiver–operating characteristics (ROC) curves of cervical length and fetal head-pubis symphysis distance measured by translabial ultrasound and Bishop Score

AUC was 0.61 (*P* = 0.04) for Bishop Score with sensitivity and specificity of 84 and 70 %, respectively. Cutoff score for Bishop Score was 5. Bishop Score had slightly lower sensitivity compared with fetal head-pubis symphysis distance. AUC for cervical length measured by translabial ultrasound was 0.62 with sensitivity of 90 %, specificity of 65 % and cutoff of 12.5 mm (Table 3 and Fig. 2). In Fig. 2 ROC curves for both modalities (defined based on mode of delivery) has been shown. As demonstrated in Fig. 2, the AUC of cervical length measured by ultrasound (0.63, CI: 0.49-0.71) is slightly higher than the AUC of Bishop Score (0.62, CI: 0.48-0.71). Both methods had admissible sensitivity and specificity in predicting mode of delivery.

**Table 3:** Ultrasound parameter and bishop score sensitivity and specificity

Parameter	AUC	Sensitivity	Specificity	Cut off	NPV	PPV	*P*-value
Bishop score	0.618	84 %	70 %	5	44 %	100 %	0.044*
Cervical length (mm)	0.628	90 %	65 %	12.5	100 %	72 %	0.034*
Fetal head-pubis symphysis distance (mm)	0.656	88 %	70 %	12	46 %	100 %	0.010*

*AUC* Area under curve, *NPV* Negative predictive value, *PPV* Positive predictive value

*Significance at the level of *P* < 0.05

## Discussion

Ultrasound examination is a safe, quick and non-invasive method that has been shown to provide valuable objective measurements for monitoring progress of a labor [27]. Traditional methods observe labor progress by frequent examinations and assessment of Bishop Score. Recently, the use of ultrasound examinations is increasing to evaluate labor. However, there are limited literatures that have compared the value of ultrasound parameters with traditional examinations by assessing Bishop Score. With increasing concerns about the accuracy of Bishop Score and its value to determine the suitability of IOL, researches have emphasized on the necessity to develop alternative assessment standards [6–11]. Previously, Eggebø et al. [3] illustrated that fetal head–perineum distance measured by trans perineal ultrasound examination is a predictor of vaginal delivery after IOL with a similar predictive value compared with ultrasound-measured cervical length and the Bishop score. However, since it is well-established that parity is a strong predictor of successful IOL [14], further investigations on homogenous populations with similar parity were required to come to a proper comparison of these methods. In this study, only primigravid women were included and our results showed that translabial ultrasound measurement can predict the suitability of IOL marginally better than Bishop Score. Test sensitivities were 88, 90 and 84 % for fetal head-pubis symphysis distance, cervical length and Bishop Score, respectively which indicates slightly better predictive value in ultrasound measurements. These findings confirm that ultrasound is a valuable non-invasive tool with a good diagnostic accuracy. By considering the low cost and reduced inter observer variability of ultrasound [28–33], this measurement tool can be recommended to be used routinely to monitor labor.

The superior predictive value of cervical length measured by trans-vaginal ultrasound compared with Bishop Score has been already demonstrated in previous studies [34–36]. However, since trans-vaginal ultrasound requires placing a vaginal probe, translabial ultrasound is better tolerated and accepted by most women. Here, we showed that translabial ultrasounds examination can also provide better and more reliable measurements for predicting labor compared with Bishop Score. Furthermore, it seems that extreme measures in both methods can predict the necessity of C/S with similar value. In this regard, Tan et al. showed that a cervical length >20 mm and bishop score <5 are independent predictors of C/S [37].

Some investigations have proposed that measurement of fetus entry angle and occiput position may provide additional predictive values [38, 39]. However, the predictive values of entry angel and occiput position have not been approved by other studies [40] which is may be due to recruitment of heterogeneous populations. To our knowledge, this is the first study on a homogenous population and our results confirm the superior value of ultrasound measurements including fetal head-pubis symphysis distance and cervical length in prediction of vaginal delivery after IOL. Furthermore, our results do not support the predictive value of entry angle and fetus occiput position on a homogenous population. Extreme measures of fetus weight including macrosomia have been shown to be associated with necessity of C/S [41] however in this study fetus weight was not a determinant of operative delivery which is due to similar range of fetus weight among women with NVD and candidates of C/S. No beneficial effect of anterior occiput position could be detected in predicting successful IOL as well. It seems that clinical models with combined consideration of maternal factors and ultrasound measurements are needed for a proper prediction of successful vaginal delivery for each individual [42, 43].

In line with Eggebø et al. [3], our investigation did not confirm the predictive value of maternal age which conflicts with some other reports [13]. One reason for this difference can be the characteristics of the study population. Here, we only included primigravid women who have relatively similar ages and are younger than multiparous women. Previous investigations have tried to identify a model for combined consideration of maternal characteristics including age to predict outcome of IOL [44]. Further investigations with inclusion of various age groups and adjustment for other maternal confounders are required to determine the effect of age on labor.

The rate of delivery by Cesarean section in our department was 43 % which is noticeably higher than Cesarean rate in Norway (13 %) [3] and is similar to Mexico (43.9 %), Italy (39.8 %) and South Korea (35.3 %) [45]. There are many factors that affect this increasing Cesarean trend including socio-economic class [46], protocol of the related hospital [47] and increased technology [48]. It seems that the characteristics of population in each nation affect the rate of C/S. Therefore, more investigations are required to determine the Iranian population features to clarify the reasons behind this high rate of C/S.

This study shows that translabial ultrasound can predict the successful vaginal delivery after induction of labor better than Bishop Score. Cervical length and fetal head-pubis symphysis distance measured by translabial ultrasounds have admissible test sensitivity of 90 % and 88 %, respectively. This method is safe, non-invasive and provides valuable objective measures to predict suitability of induction of labor. Translabial ultrasound can be better accepted by women when considering the painful process of assessing Bishop Score. Moreover, by considering the reduced risk of infection by using ultrasound measurements especially in case of rupture of the amniotic sac, translabial ultrasound is suggested to be routinely used to monitor the progress of the labor.

## Conclusion

In this investigation, the predictive values of Bishop Score and translabial ultrasound measurements in determining suitability of IOL have been evaluated. This study demonstrates that translabial measurements can be a suitable alternative method to monitor labor progress with an admissible predictive value compared with Bishop Score. It is a non-invasive method which provides valuable objective measurements and can be better accepted by women when considering the painful process which is required in evaluating Bishop Score.

### Study limitation

This study suggests that ultrasound measurement be considered as a routine examination to monitor the progression of the labor in clinical practice. Subsequently, the need for the skillful staff to perform ultrasound and to render accurate sonographic measurements increases which should be taken into consideration.
